# A *count of* coping strategies: A longitudinal study investigating an alternative method to understanding coping and adjustment

**DOI:** 10.1371/journal.pone.0186057

**Published:** 2017-10-05

**Authors:** Taylor Heffer, Teena Willoughby

**Affiliations:** Department of Psychology, Brock University, St. Catharines, Ontario, Canada; Hunter Holmes McGuire VA Medical Center, UNITED STATES

## Abstract

Researchers recently have suggested that coping flexibility (i.e., an individual’s ability to modify and change coping strategies depending on the context) may be an important way to investigate coping. The availability of numerous coping strategies may be an important precursor to coping flexibility, given that flexibility can only be obtained if an individual is able to access and use different coping strategies. Typically, studies examining the use of coping strategies compute means-based analyses, which assess not only what strategies are used but also how much they are used. Thus, there is limited ability to differentiate between individuals who use a lot of strategies infrequently, and individuals who use only one or two strategies a lot. One way to address this confound is to count the number of strategies that an individual uses without attention to how frequently they use them (i.e., a count-based approach). The present longitudinal study compares a count-based model and a means-based model of coping and adjustment among undergraduates (*N* = 1132). An autoregressive cross-lagged path analysis revealed that for the count-based approach, using a greater number of positive coping strategies led to more positive adjustment and less suicide ideation over time than using a smaller number of positive coping strategies. Further, engagement in a greater number of negative coping strategies predicted more depressive symptoms and poorer emotion regulation over time. In comparison, the means-based model revealed identical results for negative coping strategies; however, engagement in more frequent positive coping strategies did not predict better positive adjustment over time. Thus, a count-based approach offers a novel way to examine how the number of coping strategies that individuals use can help promote adjustment among university students.

## Introduction

For many students, attending university can be stressful and challenging [1,2]. Students often are faced with many demands (e.g., moving away from home, struggling with financial constraints, etc.) often without the close social support of family and friends that they experienced when living at home [3,4]. Importantly, accumulation of these daily stressors can impact students’ adjustment [2,5,6]. Indeed, the rates of suicide ideation and depressive symptoms among university students are alarming. In a study of 16,760 American undergraduates, 36.1% reported feeling so depressed in the past year that it was difficult to function and 10.3% seriously considered suicide—yet many students may not seek out or be aware of appropriate resources that are available to them [7,8]. Thus, managing these challenges places a reliance on students’ own ability to cope. The current study seeks to investigate how the number of coping strategies that individuals use may be associated with adjustment over time.

The transactional theory of coping posits that coping is an evolving process that changes in response to context, in an effort to manage different internal and external demands [9]. Accordingly, the transactional theory of coping presumes that successful coping involves an ability to adjust and change coping strategies in a way that facilitates positive outcomes.

With this in mind, current models of coping have focused on the idea of coping flexibility- a way of studying coping that identifies an individual’s ability to modify their coping behavior according to the nature of each stressful situation (see [10]).

The availability of numerous coping strategies when stressed may be an important precursor to coping flexibility—in order to demonstrate flexibility among a variety of coping strategies, individuals must first possess a diverse range of coping strategies that they are able to use when stressed [11]. Studies investigating the use of coping strategies typically compute means-based analyses whereby they not only investigate what strategies are used, but also how much (i.e., a little, a medium amount, a lot) each is used—a composite score then is computed based on the average frequency of use across all the strategies [12–15]. As a result, this approach is unable to differentiate between individuals who use a lot of strategies infrequently and individuals who use only one or two strategies a lot. For example, an individual who uses three coping strategies “a little” (scored as a 2 on the Likert scale) would have an identical mean to someone who indicates using two strategies “not at all” (scored as a 1) and a third strategy “a lot” (scored as a 4); both means would be 2. In other words, when using a means-based analysis, distinct coping patterns can present with identical means, limiting the conclusions that can be made regarding the relationship between the number of coping strategies used and adjustment. One way to address this confound is to count the number of strategies that an individual uses when stressed without attention to how frequently they use them (i.e., a count-based approach).

Regardless of approach (count or mean), it also is important to note that some strategies may not be advantageous, regardless of how well an individual is able to use that specific strategy [16]. For instance, consider a person who copes with different situations by blaming themselves, self-medicating through alcohol use, and seeking support; this person would not be expected to have a more favourable outcome compared to if they had just used only one strategy such as seeking support, given that self-blame and alcohol use are unlikely to help. Thus, adaptive coping may require an ability to use coping strategies that are at least relatively positive in nature. The current study examines this hypothesis by separating coping strategies based on positive and negative coping. In doing so, differential associations between adjustment and the count of positive strategies versus the count of negative coping strategies used can be assessed. Of note, however, there may be some instances where certain coping strategies may not be considered to be truly negative or positive (e.g., distraction coping may not help an individual succeed on an exam). Thus, we acknowledge that these terms may be oversimplified.

### Coping and negative adjustment

Despite the potential benefits of using multiple strategies to cope with stress, doing this may be difficult for individuals experiencing poor adjustment. Two indicators of poor adjustment that are examined in the current study are depressive symptoms and suicide ideation. Importantly, individuals with high levels of depressive symptoms demonstrate a more negative attribution style (i.e., a stable and internalized attitude that unpleasant circumstances will persist) compared to their non-depressed peers ([17,18]; see [19] for a review). Thus, believing that nothing can be done to alter an aversive situation may discourage an individual from seeking out new positive ways to cope with problems.

In line with this idea, concurrent studies using a means-based approach have found that using more frequent negative coping strategies (e.g., self-blame) are associated with higher depressive symptoms [20]. Further, in a longitudinal investigation, Lee and colleagues [21] found that more frequent engagement in avoidant coping was associated with more depressive symptoms over time, although they only tested one direction—from coping to depressive symptoms over time (see also [22]). Thus, interpretation of these findings generally is that negative coping leads to more depressive symptoms over time. However, a longitudinal study testing bidirectionality is necessary before conclusions about the direction of effects can be ascertained.

Suicide ideation also is associated with how well individuals are able to cope with stress [23–26]. For example, findings from concurrent studies indicate that individuals with higher levels of suicide ideation engage in more frequent (calculated by a means-based approach) maladaptive coping strategies [23, 25] and tend to have more trouble problem solving in the face of stress [27], compared to individuals with lower levels of suicide ideation. Thus, individuals who engage in more suicide ideation may have more difficulty accessing multiple productive coping strategies when faced with stress. But it also may be that individuals who use more negative coping strategies in the face of stress have higher suicide ideation over time- a longitudinal study testing both directions of the effects is required in order to address these hypotheses.

Overall, while there is evidence of a means-based association between coping and negative adjustment, less is known about whether these results are transferable when looking solely at the number of strategies individuals have available to them. Interestingly, researchers often suggest that one way to help decrease negative adjustment (e.g., depressive symptoms and suicide ideation) may be to reduce the *number* of negative coping strategies that individuals use. Yet, a direct test of this hypothesis has not been conducted. Research examining a count-based approach is necessary before concluding that the number of strategies that individuals use is associated with adjustment. In addition, the current study will investigate the direction of effects of these relations over time. For example, it may be that individuals who engage in a greater number of negative coping strategies when stressed report more depressive symptoms and suicide ideation over time than their peers. On the other hand, individuals who report depressive symptoms and suicide ideation at Time 1 may engage in a greater number of negative coping strategies over time. In fact, both possibilities may be true—the effect may be bidirectional. Thus, an important goal of the present study is to investigate the direction of effects of these relations for both positive and negative coping.

### Coping and positive adjustment

A second objective of the current study is to investigate the relationship between coping and positive adjustment. Coping often is investigated in terms of its ability to decrease negative outcomes. The current study, however, seeks to investigate whether coping can also play an important role in increasing positive outcomes. Indeed, having a higher number of positive coping strategies available in the face of stress may provide the individual with more resources to deal with stress. This may allow an individual to manage stress more effectively and be more confident in their ability to deal with problems. There is less research directly investigating coping and positive adjustment than coping and negative adjustment, and the research that has been done generally is concurrent rather than longitudinal.

As adjustment can be examined in a variety of ways, in the present study we will focus on three indicators: emotion regulation, self-esteem and academic achievement. All three are associated with coping [28–30] and represent particularly important indicators of adjustment among students. One indicator of positive adjustment that is associated with coping is emotion regulation. Individuals who are better able to regulate their emotions and engage in more positive affect may be more likely to seek out and use a number of positive coping strategies. According to the broaden-and-build theory, the experience of positive emotions (e.g., joy) broadens attention and thinking (i.e., heightens openness to new possibilities, big picture focus, etc.), in comparison to negative emotions, which tend to result in a narrowing of focus (i.e., fight or flight, etc., [31]). This broadening of attention is hypothesized to build personal resources, such as adaptive coping strategies [32]. In light of this, individuals who are better able to regulate emotions in a more positive manner may have a heightened ability to think more broadly, allowing for engagement in a *variety* of positive coping strategies, compared to those who have more narrow thinking. Alternatively, it may be that individuals who engage in a greater number of positive coping strategies may have a greater sense of control, and demonstrate a greater ability to adjust their coping responses and adapt to stress. As a result, these individuals may become more proficient in their ability to regulate their emotions. Fredrickson [33] posits that both interpretations warrant investigation (i.e., bidirectionality) by emphasizing that experience of positive emotion should help facilitate a broader source of coping, which could help improve later experiences of positive emotions.

Another way that coping has been implicated in promotion of positive adjustment is in its association to self–esteem [21]. For instance, individuals with high self-esteem are thought to have more effective and appropriate coping resources available to deal with stress (e.g., planning and problem solving [34]. With regards to the count of coping strategies used, it could be that individuals with high self-esteem may be more confident in their ability to cope with different stressors (and thus be more likely to engage in a variety of positive coping strategies). It also may be, however, that individuals who are able to use a greater number of positive coping strategies may develop a sense of control and feelings of confidence in their ability to handle different situations appropriately, thus leading to increased self-esteem.

In addition, academic achievement may be another important factor associated with coping. Academic achievement typically requires an ability to work well under pressure (e.g., writing exams, oral presentations) as well as an ability to collaborate on group projects. The ability to cope efficiently and employ more frequent positive orientated strategies when under stress/pressure has been found to be associated with better academic achievement, compared to those who rely on less effective coping strategies [35, 36]. Further, Zeidner [37] emphasizes that success on exams is associated with a combined use of multiple strategies (i.e., increase study time, seek support from friends. While associations have been found between academic achievement and use of effective coping strategies, less is known about the longitudinal association between academic achievement and a count of the number of coping strategies used.

The current study seeks to investigate relationships between positive adjustment and a count of the number of strategies individuals use. A count-based analysis will help to clarify if having a number of positive coping strategies available when stressed will be associated with positive adjustment over time. It also is necessary to use a longitudinal design in order to assess bidirectionality. As an example, interpretations of concurrent studies surrounding academic achievement imply that having better coping strategies leads to better academic achievement; it also could be, however, that the ability to succeed in an academic setting may help build confidence and lead to a broadening of focus which could help increase the use of a variety of coping strategies. The same issues with interpretations can be applied to emotion regulation and self-esteem, thus further longitudinal examination is required.

### Stress as a moderator

While a key goal of the present study is to investigate bidirectionality, it is quite possible that the associations among these variables may differ depending on the individuals’ level of stress. For instance, coping is generally considered in the context of stress; thus if an individual is *not* experiencing stress, we might not expect them to apply and use a number of coping strategies compared to individuals who are experiencing stress [38,39]. Indeed, it may be that individuals who have a lot of different stressors in their life may benefit more from using a variety of strategies, compared to individuals who have few stressors.

### The current study

There are three main research questions associated with this longitudinal study. First, how is a count-based approach associated with adjustment over time, and are these effects bidirectional? Although research using a means-based approach has provided evidence for associations between coping and adjustment, little work has used a count-based approach or used this approach with a longitudinal design. We predict that using a greater number of positive coping strategies when stressed might be associated with better adjustment (i.e., less depressive symptoms, less suicide ideation, more self-esteem, better emotion regulation and higher academic achievement) over time than using a smaller number of positive coping strategies. We also expect that using a higher number of negative strategies will be associated with poorer adjustment (e.g., greater depressive symptoms, and higher suicide ideation) than using a smaller number of negative coping strategies. Given the lack of research, it is not clear whether using a greater number of negative coping strategies will be associated with poorer self esteem, emotion regulation and academic achievement over time. Further, the analyses examining bidirectionality in these associations over time are exploratory.

Second, the current study offers a comparison of a count-based approach and a means-based approach to studying coping and adjustment. Given that a counts-based model does not take into consideration how much individuals use each strategy and only examines the number of coping strategies individuals use, it also would be beneficial to compare this model to a means-based model that takes both of these factors into consideration. In doing so, differential associations between the two models can be compared in order to address the ways in which a count-based approach may be an alternative method to studying coping.

A third purpose of this study is to investigate whether stress is an important moderator of the association between coping (for both the count-based and the means-based methods) and adjustment. Additionally, all analyses controlled for sex and parental education given research suggesting that these variables are associated with coping and adjustment [40–42].

## Method

### Participants

The current sample of 1,132 (70.5% female) first-year undergraduate students (*M*_age_ = 19.06, SD = .92) from a mid-sized Canadian university was drawn from a larger longitudinal study examining adjustment in university. In total, 87.5% of the participants were born in Canada. Consistent with the broader demographics for the region; the most common ethnic backgrounds endorsed other than Canadian were British (19%), Italian (16.8%), French (9.5%) and German (9%; [43]). Data on socioeconomic status indicated mean levels of parental education falling between “some college, university or apprenticeship program” and “completed a college/ apprenticeship/ technical diploma.”

Missing data occurred within each assessment time point because some students did not finish the entire questionnaire (average missing data = 1.8%) and because some students did not complete both waves of the data. Out of the original sample that completed the survey at Time 1, 73.1% completed Time 2 of the survey. The overall multivariate test for missingness was significant, *Λ* = .941, *F*(9, 1010) = 7.017, *p* < .001, η^2^ = .059. Participants who were missing at the second time point were not significantly different from participants who were there at both time points, with two exceptions. Specifically, those who completed both waves of the study were more likely to be females and to have higher grades compared to those who only completed one wave of the study (*p*s < .001). Missing values were imputed using the expectation–maximization algorithm (EM; iterations = 200) with all study measures included in the analysis, thus avoiding the biased parameter estimates that can occur with pairwise deletion, list-wise deletion or means substitution [44].

### Procedure

First-year university students were invited to participate in the survey examining factors related to stress and adjustment. The study was advertised by way of posters, emails, classroom announcements, website posting, and residence visits. Students could participate regardless of academic major, and were given monetary compensation or course credit for their participation. Only students who completed the first wave were invited (by email and/or phone) to participate again in the second wave. The Social Science Research Ethics Board approved the study (Ethics Approval Number: 09–118) and all participants provided informed written consent. Trained research assistants administered the survey. To ensure the safety of our participants a full debriefing was provided at the end of the survey and a list was given of both available mental health resources and researcher contact information. Participants also were given the opportunity during the survey to provide their contact information so that they could be contacted by a mental health professional if they were experiencing any distress.

### Measures

#### Demographics

Sex and parental education (one item per parent, scale ranged from 1 (*did not finish high school*) to 6 (*professional degree*), averaged for participants reporting on both parents; *r* = .40) were assessed at Time 1.

#### Coping

Coping was assessed using a shortened version of the Brief COPE (15 items) at Time 1 and then again one year later at Time 2 [45]. The Brief COPE includes positive and negative coping strategies. In order to differentiate between these positive and negative coping strategies, a principal components factor analysis with direct oblimin rotation was conducted using the data from Time 1. Four components emerged with eigenvalues > 1. Factor 2 was comprised of four negative coping items that hung together (i.e., self-blame, self-criticism, alcohol use, and giving up; eigenvalues = 2.73) with factor loadings ranging from 0.63 to 0.77. These items thus were included in the count of negative coping strategies. The three remaining factors reflected different subtypes of positive coping strategies such as religion (e.g., I pray or meditate), seeking support (e.g., I get emotional support from others), and reframing/humor (e.g., I look for something good in what is happening). Indeed, previous research has found that positive adjustment is associated with positive reframing and humor [46], seeking support [47] as well as religious coping strategies (see [48]). As the focus of this study was to investigate how many strategies individuals have access to using (regardless of the subtype of positive strategies), the items from the three remaining factors were grouped together in order to create the count of positive coping strategies (see S1 Table for more information on the factors).

When filling out the coping measure, participants were asked to indicate what they do when under a lot of stress on a scale ranging from 1 (*I usually don’t do this at all*) to 4 (*I usually do this a lot*). In order to create a count of how many strategies individuals use when stressed, the items were recoded such that that 0 represented not using the strategy (i.e., *I usually don’t do this at all*), while 1 represented using the strategy to any degree (i.e., *I usually do this a little bit*, *I usually do this a medium amount*, *I usually do this a lot*).

The count of negative coping strategies was created by counting the number of negative strategies individuals use when stressed (e.g., “I blame myself”, “I use alcohol and other drugs to make myself feel better,” etc.). An average of these strategies (based on the original items with the four-point scale) was also created and used in the means-based approach. Cronbach’s alpha was .68 at Time 1 and .72 at Time 2. The count of positive coping strategies was assessed by counting the number of positive strategies individuals use when stressed (e.g., “I get comfort and understanding from someone,” “I look for something good in what is happening” etc.). An average of these strategies (based on the original items with the four-point scale) was also created and used in the means-based approach. Cronbach’s alpha was .76 at Time 1 and .74 at Time 2. The Brief COPE has been shown to have good internal consistency and validity in previous research [45].

#### Depressive symptoms

Participants completed The Center for Epidemiological Studies Depression Scale at Time 1 and Time 2 in order to assess their level of depressive symptoms (CES-D Scale; [49]; e.g., “I felt lonely” and “My sleep was restless”). Individuals indicated on a scale of 1 (*none of the time*) to 5 (*most of the time*) how often they experienced 20 symptoms associated with depression. Cronbach’s alpha in the present study was .91 at Time 1 and .92 at Time 2.

#### Suicide ideation

Suicide ideation in the past year was assessed at Time 1 and Time 2 using a question from the Suicide Behaviors Questionnaire-Revised (SBQR; [50]; “How often have you thought about killing yourself in the past year?”). This item was rated using a 5-point scale that ranged from 1 (*never*) to 5 (*very often*). The SBQR has been shown to have good internal consistency and validity in previous research [50].

#### Self esteem

Self-esteem was measured at Time 1 and Time 2 using the Rosenberg Self-Esteem Scale [51]. The measure included 10 items (e.g., “I take a positive attitude toward myself”) that were rated on a scale from 1 (*strongly disagree*) to 5 (*strongly agree*). Cronbach’s alpha was .904 at Time 1 and .916 at Time 2.

#### Academic achievement

Academic achievement was measured at both Time 1 and Time 2 using students’ academic average for the corresponding year, recorded in percentages (e.g., 70%). Information was obtained from the University Registrar with the participants’ permission.

#### Emotion regulation

Emotion regulation was assessed at both Time 1 and Time 2 using 6 items from the Difficulties in Emotion Regulation (DERS; [52]); e.g., ‘‘When I’m upset or stressed, I have difficulty concentrating”). The responses were based on a five-point Likert scale ranging from 1 (*almost never*) to 5 (*almost always*). The scale was recoded so that higher scores indicated better emotion regulation. Cronbach’s alphas at Time 1 and Time 2 were .73 and .74, respectively.

#### Stress

Stress was measured using The Daily Hassles Scale. Participants indicated how bothered they felt by 25 daily hassles. Hassles related to daily life stressors such as peer conflict, family, school and money (e.g., “Being lonely” and “Not having enough time”). Responses were rated on a scale from 1 (*almost never bothers me*) to 3 (*often bothers me*). Cronbach’s alpha for these 25 items was .84.

## Results

### Preliminary analyses

The means and standard deviations of all study variables are outlined in Table 1. All variables demonstrated acceptable levels of skewness and kurtosis with the exception of suicide ideation, which was transformed using the log-likelihood method to correct for non-normality. There was a significant main effect of sex on the number of positive coping strategies used, with females reporting using a greater number of positive coping strategies than males at both Time 1 and Time 2, *p*s < .004. Females also reported having more depressive symptoms than males at Time 1, *p* < .001, and higher academic achievement at Time 2, *p* = .006, than males. In contrast, males were significantly more likely to have better emotion regulation than females at both Time 1 and Time 2, *ps* < .001. At Time 2, males were more likely to engage in a greater number of negative coping strategies, *p* = .027, and also reported higher suicide ideation, *p* = .014, than females. There were no significant differences on parental education, *p* > .05.

**Table 1:** Means and standard deviations for all study variables.

Variables	Time 1 *M* (*SD*)	Time 2 *M* (*SD*)
**Positive Coping (Count)**	8.164 (2.110)	8.288 (1.813)
**Negative Coping (Count)**	2.351 (1.259)	2.342 (1.182)
**Positive Coping (Mean)**	2.344 (0.499)	2.381 (0.431)
**Negative Coping (Mean)**	1.939 (0.652)	1.910 (0.596)
**Depressive Symptoms**	2.115 (0.647)	2.090 (0.619)
**Suicidal Ideation**	1.391 (0.845)	1.367 (0.726)
**Emotion Regulation**	3.214 (0.733)	3.148 (0.694)
**Academic Achievement**	67.375 (11.114)	68.065 (11.425)
**Self-Esteem**	3.806 (0.688)	3.811 (0.676)
**Stress**	1.927 (0.319)	
**Sex (%)**	70.5% Female	
**Parental Education**	3.654 (1.267)	

### Primary analyses

**Fig 1:**
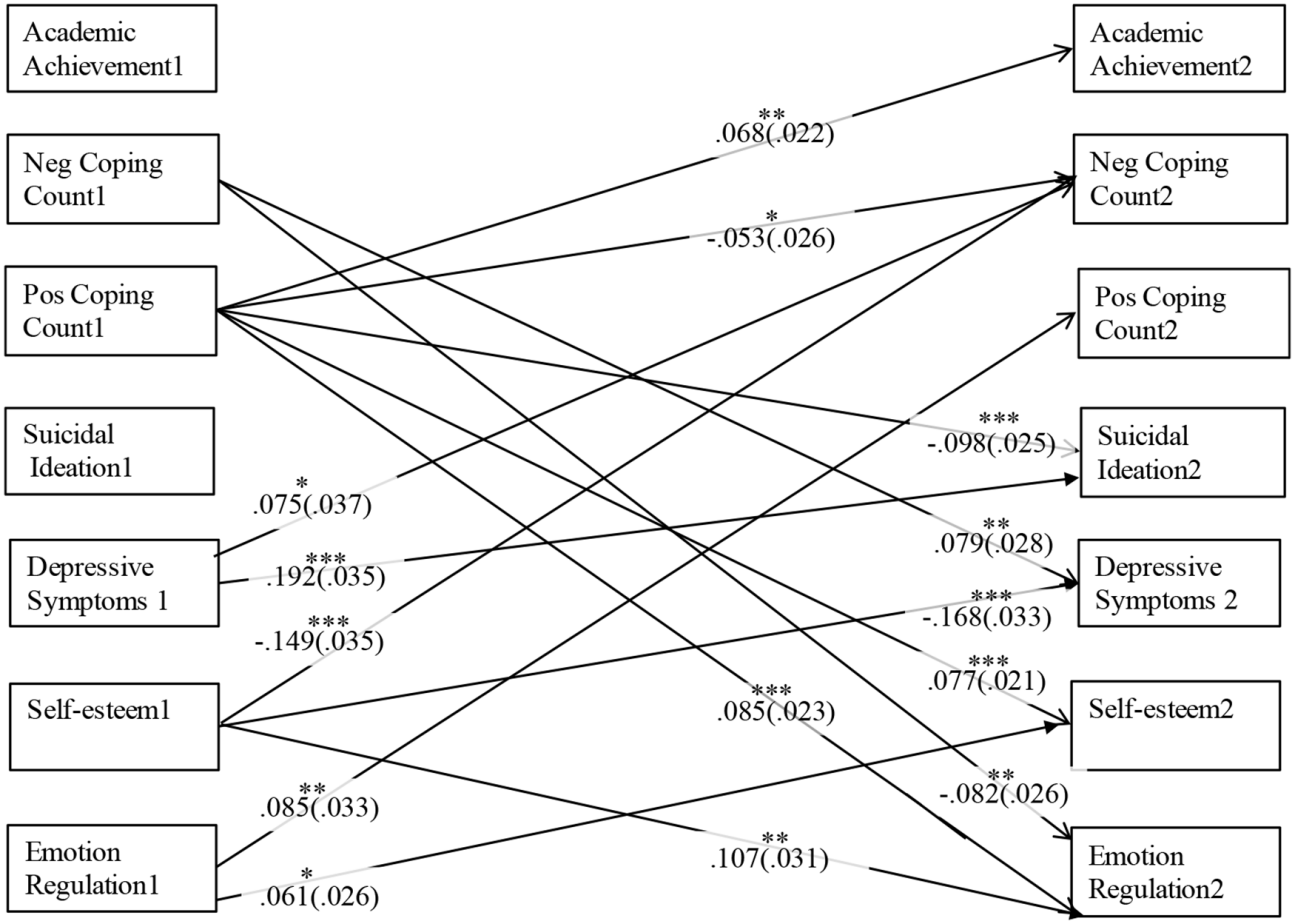
Significant cross-lagged paths between all key study variables for the count-based model. Numbers 1 and 2 indicate Time 1 and Time 2, respectively. Values indicate standardized beta weights (standard errors are in parenthesis). Pos = Positive, Neg = Negative.

**Fig 2:**
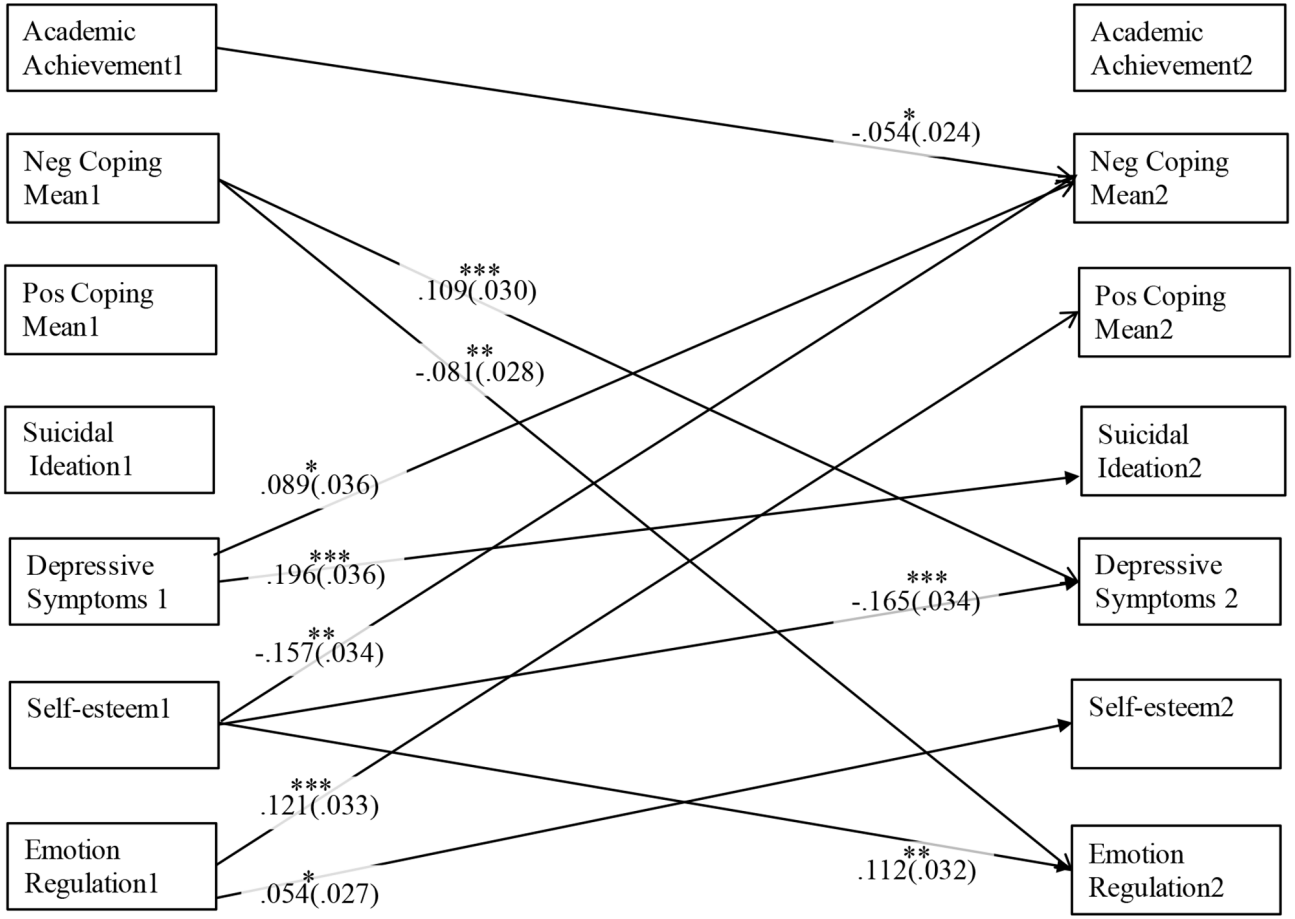
Significant cross-lagged paths between all key study variables for the means-based model. Numbers 1 and 2 indicate Time 1 and Time 2, respectively. Values indicate standardized beta weights (standard errors are in parenthesis). Pos = Positive, Neg = Negative.

The primary statistical analyses were carried out using an auto-regressive cross-lagged path analysis in MPlus 7. Two models were run, a count-based model and a means-based model. The models were comprised of seven variables measured over 2 years: positive coping strategies, negative coping strategies, depressive symptoms, suicide ideation, academic achievement, emotion regulation, and self-esteem (see Figs 1 and 2). Across the two time periods, we included cross-lag paths among all seven key study variables, autoregressive paths (i.e., within each variable), and concurrent associations among all variables within each wave. Sex and parental education also were included as covariates, such that correlations were specified between each of the covariates and each variable at Time 1 and paths were estimated between the covariates and each variable at Time 2. Any significant path, therefore, accounted for covariates, previous scores on the outcome variables, correlations among variables within a wave, as well as any other predictors in the model (i.e., estimating the unique relation between study variables). Significant paths among the seven key study variables for both models (count-based and means-based) are depicted in Figs 1 and 2 (see S2 and S3 Tables for full results among key variables). Model fit was not relevant given that the models were saturated.

The following results for the paths related to coping were consistent across both the count-based model and the means-based model (See Figs 1 and 2 as well as S2 and S3 Tables for specific path results as well as all results among adjustment indicators). There was a bidirectional association between the use of negative coping strategies and depressive symptoms, such that using more (as measured by a count and a mean) negative coping strategies at Time 1 was associated with higher depressive symptoms at Time 2, and depressive symptoms at Time 1 were positively associated with more engagement in negative coping strategies at Time 2. There also was a unidirectional association found between the use of negative coping and emotion regulation; specifically, using less negative coping strategies (as measured by a count and a mean) at Time 1 was associated with better emotion regulation at Time 2.

Critically, some results were not consistent among the two models. For the count-based model, using a greater number of positive coping strategies at Time 1 was associated with less suicide ideation, engagement in fewer negative coping strategies, higher self-esteem, as well as higher academic achievement one year later. There was also a bidirectional association between the number of positive coping strategies used and emotion regulation. Using a greater number of positive coping strategies at Time 1 was associated with better emotion regulation at Time 2, and better emotion regulation at Time 1 was associated with use of a greater number of positive coping strategies at Time 2.

For the means-based analysis, in addition to the overlapping findings among both models, there also was a unidirectional association found between positive coping and emotion regulation, such that better emotion regulation at Time 1 was associated with more positive coping (means-based) at Time 2. Further, there was a unidirectional association between academic achievement and negative coping. Specifically, higher academic achievement at Time 1 was associated with less negative coping (means-based) at Time 2.

We assessed whether stress was a significant moderator of the pattern of results in both the count-based and means-based models. Stress was categorized into two equal percentiles (50% each) encompassing higher versus lower daily stress. The Chi-Square Difference Test of Relative Fit was not significant for either the count model, χ^2^_diff_(42) = 45.516, *p* = .292, or the means-based model χ^2^_diff_(42) = 42.727, *p* = .439, indicating that the pattern of associations for both models was not different between people with lower stress compared to people with higher stress. We also assessed whether stress might be a significant moderator if we only included individuals who scored at the more extreme ends of stress (bottom 33% vs top 33%). Consistent with the previous result, the Chi-Square Difference Test of Relative Fit was not significant for either the count-based model, χ^2^_diff_(42) = 25.439, *p* = .980 or for the means-based model χ^2^_diff_(42) = 27.275, *p* = .961. Overall, these results reveal that stress does not appear to be a moderator of the pattern of results between coping and adjustment.

## Discussion

A large volume of research has been conducted on coping, stress, and adjustment [53]. In line with the transactional theory of coping, coping flexibility is an important way of studying coping that accounts for an individual’s ability to adjust and change coping styles in response to different internal and external demands [9]. Importantly, the availability of numerous coping strategies may be an important precursor to coping flexibility, given that flexibility may only be obtained if an individual is able to access and use different coping strategies [11]. Studies that have investigated the use of coping strategies, however, typically compute a means-based analysis—an approach that does not allow for differentiation between individuals who use a lot of strategies infrequently and individuals who use only one or two strategies a lot. In order to address this limitation, the current study created a count-based measure of coping, whereby the number of strategies that an individual uses was counted without attention to how frequently they use them.

The focus of the present study was to investigate the relationship between a count-based approach to coping and adjustment. Critically, using a greater number of positive coping strategies was associated with better adjustment (e.g., less suicide ideation, using a fewer number of negative coping strategies, higher self-esteem and better academic achievement) over time. Of note, this finding was not true for the means-based analysis. This is an important finding as it suggests that encouraging students to use a greater number of positive coping strategies can not only help to decrease negative adjustment, but also aid in promoting positive adjustment.

In terms of bidirectionality, there was a bidirectional relationship between using a greater number of negative coping strategies and more depressive symptoms. This finding is in line with the research suggesting that individuals with depression may have a more negative attribution style and thus may be more likely to use strategies such as giving up. Additionally, using these types of negative coping strategies predicted more depressive symptoms over time. In line with the broaden-and-build theory, a bidirectional association also was found between emotion regulation and the number of positive coping strategies used when stressed. Our results suggest that emotion regulation may be a distinct way to help broaden an individual’s positive coping resources when stressed, and in turn, individuals who use a greater amount of positive coping strategies when stressed may be better able to regulate their emotions in a more positive manner.

Another goal of the current study was to compare a means-based approach to a counts-based approach. Overall, it appears that the count-based approach offers similar findings to the means-based approach in terms of negative coping. The count-based approach, however, provided additional findings that suggest that using a greater number of positive coping strategies may be distinctly important for promoting positive adjustment as well as decreasing negative adjustment. Further research is needed to investigate why using a greater number of positive coping strategies may be adaptive. For instance, it could be that having more resources available or alternative ways to deal with stress allows individuals to deal with problems more effectively. It also is important for future research to identify the factors that lead some individuals to use more coping strategies than their peers (e.g., access to role models, higher executive functioning and planning skills, openness to experience, etc.). In addition, future research would benefit from identifying if there are differences between the number of strategies individuals think they might use in a situation (e.g., using hypothetical scenarios) compared to the number of strategies that they actually use when faced with stress. This would help identify whether individuals have certain strategies *available* but do not use them. Studies addressing these issues could help inform interventions aimed at teaching individuals how to use a variety of positive coping strategies as a way to promote adjustment.

The current study also found that stress was not a significant moderator of the relation between coping strategies and adjustment. This finding suggests that the using a greater number of positive coping strategies as well as using less negative coping strategies (lower average and a fewer number of negative strategies) may be beneficial for people with either high or low stress. Thus, even if an individual does not have a lot of stress in their life, it is still beneficial to have a greater number of positive coping strategies available to deal with problems effectively.

This study has important strengths, including a large sample, multiple indicators of adjustment, as well as being the first longitudinal study to offer a comparison between a means-based approach and a count-based approach to coping and adjustment. At the same time, the study has several limitations. First, generalizability is limited due to a predominantly Caucasian sample of university students. Second, the measure of stress comes from a self-report questionnaire of daily hassles. Thus, this measure is targeting more minor daily stressors, compared to major or severe stressors. It is worth noting, however, research findings emphasize the importance of cumulative daily stress/hassles in the role of negative adjustment [54,55]. Nonetheless, future research may benefit from investigating if the relationship between the number of coping strategies used and adjustment is more prominent among individuals facing major stressors. Another limitation is that coping was assessed via retrospective reports. It would be valuable for future research to assess these constructs in real time through techniques such as ecological moment sampling (e.g., daily diaries). Of note, the current study was unable to assess how coping may change depending on the situational context. Admittedly, it would be extremely difficult to evaluate and account for varying subjective stressors, as well as dispositional and environmental factors, in order to identify an objective measure of how coping may be adaptive in response to *specific* contexts [56]. Future research is needed to help disentangle how context may play a role in the relationship between a count of coping strategies used and adjustment.

### Conclusion

In conclusion, the present study helps to elucidate the associations between adjustment and two methods of investigating coping over time. Understanding coping behaviours over time can help researchers and practitioners implement programs to improve coping efficiency and adjustment. Studies that investigate only a means-based approach are unable to differentiate between individuals who use one or two strategies a lot as opposed to those who use multiple strategies infrequently. Thus, a count-based method offers an innovative and practical way to implement interventions that could focus on teaching individuals to use a larger variety of coping strategies. Indeed, using a greater number of positive coping strategies is associated with less use of negative coping strategies, less suicide ideation, as well as higher self-esteem, emotion regulation, and academic achievement over time. Further, decreasing the ways in which individuals use negative coping strategies (average and count), can help to decrease depressive symptoms as well as increase emotion regulation over time. Given that university students report alarming rates of depressive symptoms and suicide ideation [8], there is a strong need for research investigating ways to decrease mental health problems as well as promote more positive adjustment.

## Supporting information

S1 Table Exploratory factor analysis. (DOCX)

S2 Table Autoregressive cross-lagged results for the count-based model. (DOCX)

S3 Table Autoregressive cross-lagged results for the means-based model. (DOCX)
