# Supplementary material for: A count of coping strategies: A longitudinal study investigating an alternative method to understanding coping and adjustment
Source: PLoS One. 2017 Oct 5;12(10):e0186057. doi: 10.1371/journal.pone.0186057 (PMC5642021; doi:10.1371/journal.pone.0186057)
Supplement: S1 Table — (DOCX) [file pone.0186057.s001.docx]

| Items | Loadings |  |  |  |  |
| --- | --- | --- | --- | --- | --- |
|  | Factor 1 | Factor 2 | Factor 3 | Factor 4 |  |
| I make fun of the situation | 0.839 | 0.109 | 0.127 | -0.040 |  |
| I look for something good in what is happening | 0.715 | -0.128 | -0.092 | 0.245 |  |
| I make jokes about it | 0.836 | 0.048 | 0.050 | -0.068 |  |
| I try to see it in a different light, to make it seem more positive | 0.706 | -0.212 | -0.092 | 0.122 |  |
| I blame myself for things that have happened | -0.196 | **0.765** | -0.158 | 0.036 |  |
| I use alcohol or other drugs to make myself feel better | 0.193 | **0.631** | 0.228 | -0.005 |  |
| I criticize myself | -0.161 | **0.699** | -0.151 | 0.151 |  |
| I give up on trying to deal with it | 0.034 | **0.727** | 0.111 | -0.005 |  |
| I try to get advice or help from other people about what to do | -0.005 | 0.047 | -0.869 | -0.014 |  |
| I get comfort and understanding from someone | -0.028 | 0.032 | -0.786 | 0.118 |  |
| I get emotional support from others | 0.003 | -0.015 | -0.855 | -0.008 |  |
| I try to find comfort in my religious or spiritual beliefs | 0.045 | 0.063 | -0.051 | 0.902 |  |
| I pray or meditate | 0.071 | 0.082 | 0.026 | 0.914 |  |
| I turn to work or other activities to take my mind off things | 0.352 | 0.024 | -0.344 | -0.026 |  |
| I do something to think about it less, such as going to the movies, watch TV, read, daydream, sleep | 0.295 | 0.305 | -0.233 | -0.155 |  |
|  |  |  |  |  |  |
| Eigenvalue | 3.364 | 2.273 | 2.000 | 1.556 |  |
| % of variance | 22.433 | 15.152 | 13.331 | 10.373 |  |

Exploratory factor analysis results (pattern matrix) for the coping items using principle components and oblique (oblimin) rotation

*Note.* There were two distraction items that did not fit with the 4 factors. We ran two models, one including and one not including these two items to ensure that they did not affect the results. The only finding that changed was that, in the means-based model, academic achievement no longer predicted negative coping when the distraction items were not included. Given that this finding was not central to our study, we elected to keep the model with the distraction items included.
