# Supplementary material for: A count of coping strategies: A longitudinal study investigating an alternative method to understanding coping and adjustment
Source: PLoS One. 2017 Oct 5;12(10):e0186057. doi: 10.1371/journal.pone.0186057 (PMC5642021; doi:10.1371/journal.pone.0186057)
Supplement: S2 Table — (DOCX) [file pone.0186057.s002.docx]

**Autoregressive Cross Lagged Results for the Count-Based Model.**

|  | B | β | | SE | 95% CI |
| --- | --- | --- | --- | --- | --- |
| Positive Coping Count1→Positive Coping Count2 | 0.411 | 0.478 | *** | 0.024 | [.432, .525] |
| Positive Coping Count1→Negative Coping Count2 | -0.029 | -0.053 | * | 0.026 | [-.103, -.002] |
| Positive Coping Count1 →Depressive Symptoms2 | -0.010 | -0.033 |  | 0.024 | [-.081, .015] |
| Positive Coping Count1→Suicidal Ideation2 | -0.008 | -0.098 | *** | 0.025 | [-.146, -.050] |
| Positive Coping Count1→Emotion regulation2 | 0.028 | 0.085 | *** | 0.023 | [.040, .130] |
| Positive Coping Count1→Self-esteem2 | 0.025 | 0.077 | *** | 0.021 | [.036, .119] |
| Positive Coping Count1→Academic Achievement2 | 0.387 | 0.068 | ** | 0.022 | [.024, .112] |
| Negative Coping Count1→Positive Coping Count2 | 0.035 | 0.025 |  | 0.030 | [-.035, .084 |
| Negative Coping Count1→Negative Coping Count2 | 0.389 | 0.414 | *** | 0.028 | [.359, .469] |
| Negative Coping Count1→Depressive Symptoms2 | 0.039 | 0.079 | ** | 0.028 | [.024, .133] |
| Negative Coping Count1→Suicidal Ideation2 | 0.001 | 0.006 |  | 0.028 | [-.049, .061] |
| Negative Coping Count1→Emotion Regulation2 | -0.045 | -0.082 | ** | 0.026 | [-.133, -.030] |
| Negative Coping Count1→Self-esteem2 | -0.008 | -0.014 |  | 0.024 | [-.061, .033] |
| Negative Coping Count1→Academic Achievement2 | -0.122 | -0.013 |  | 0.025 | [-.062, .036] |
| Depressive Symptoms1 →Positive Coping Count2 | -0.025 | 0.009 |  | 0.038 | [-.067, .084] |
| Depressive Symptoms1 →Negative Coping Count2 | 0.137 | 0.075 | * | 0.037 | [.002, .148] |
| Depressive Symptoms1 →Depressive Symptoms2 | 0.384 | 0.401 | *** | 0.034 | [.334, .467] |
| Depressive Symptoms1 →Suicidal Ideation2 | 0.049 | 0.192 | *** | 0.035 | [.123, .262] |
| Depressive Symptoms1 →Emotion Regulation2 | -0.055 | -0.052 |  | 0.033 | [-.117, .013] |
| Depressive Symptoms1 →Self-esteem2 | -0.047 | -0.045 |  | 0.030 | [-.105, .014] |
| Depressive Symptoms1 →Academic Achievement2 | 0.007 | 0.000 |  | 0.032 | [-.062, .063] |
| Suicidal Ideation1 → Positive Coping Count2 | 0.027 | 0.003 |  | 0.028 | [-.052, .058] |
| Suicidal Ideation1 → Negative Coping Count2 | -0.207 | -0.032 |  | 0.027 | [-.086, .022] |
| Suicidal Ideation1 → Depressive Symptoms2 | 0.118 | 0.035 |  | 0.026 | [-.016, .085] |
| Suicidal Ideation1 → Suicidal Ideation2 | 0.420 | 0.467 | *** | 0.024 | [.420, .514] |
| Suicidal Ideation1 → Emotion Regulation2 | 0.160 | 0.042 |  | 0.024 | [-.006, .090] |
| Suicidal Ideation1 → Self-esteem2 | -0.022 | -0.006 |  | 0.022 | [-.050, .038] |
| Suicidal Ideation1 → Academic Achievement2 | -1.140 | -0.017 |  | 0.023 | [-.063, .029] |
| Emotion Regulation1 → Positive Coping Count2 | 0.212 | 0.085 | ** | 0.033 | [.020, .150] |
| Emotion Regulation1 → Negative Coping Count2 | -0.030 | -0.019 |  | 0.032 | [-.082, .045] |
| Emotion Regulation1 → Depressive Symptoms2 | -0.040 | -0.048 |  | 0.031 | [-.107, .012] |
| Emotion Regulation1 → Suicidal Ideation2 | 0.002 | 0.110 |  | 0.031 | [-.050, .071] |
| Emotion Regulation1 → Emotion Regulation2 | 0.503 | 0.530 | *** | 0.026 | [.478, .582] |
| Emotion Regulation1 → Self-esteem2 | 0.056 | 0.061 | * | 0.026 | [.009, .112] |
| Emotion Regulation1 → Academic Achievement2 | 0.304 | 0.018 |  | 0.028 | [-.036, .073] |
| Self-esteem1 → Positive Coping Count2 | -0.037 | -0.014 |  | 0.036 | [-.085, .057] |
| Self-esteem1 → Negative Coping Count2 | -0.256 | -0.149 | *** | 0.035 | [-.218, -.080] |
| Self-esteem1 → Depressive Symptoms2 | -0.151 | -0.168 | *** | 0.033 | [-.232, -.103] |
| Self-esteem1 → Suicidal Ideation2 | -0.006 | -0.027 |  | 0.034 | [-.093, .038] |
| Self-esteem1 → Emotion Regulation2 | 0.108 | 0.107 | ** | 0.031 | [.045, .168] |
| Self-esteem1 → Self-esteem2 | 0.639 | 0.651 | *** | 0.026 | [.601, .701] |
| Self-esteem1 → Academic Achievement2 | 0.216 | 0.012 |  | 0.030 | [-.047, .071] |
| Academic Achievement1→ Positive Coping Count2 | 0.003 | 0.020 |  | 0.026 | [-.032, .071] |
| Academic Achievement1 → Negative Coping Count2 | -0.005 | -0.046 |  | 0.026 | [-.096, .004] |
| Academic Achievement1 → Depressive Symptoms2 | -0.001 | -0.024 |  | 0.024 | [-.071, .024] |
| Academic Achievement1 → Suicidal Ideation2 | 0.000 | -0.022 |  | 0.024 | [.070, .026] |
| Academic Achievement1 → Emotion Regulation2 | 0.001 | 0.019 |  | 0.023 | [-.026, .063] |
| Academic Achievement1 → Self-esteem2 | 0.002 | 0.036 |  | 0.021 | [-.005, .077] |
| Academic Achievement1 → Academic Achievement2 | 0.776 | 0.716 | *** | 0.016 | [.685, .748] |

*Note.* β *=* standardized beta weights; B = unstandardized beta weights; *SE =* standard error, CI = standardized confidence intervals. Numbers 1 and 2 indicate Time 1 and Time 2, respectively. **p* < .05. ***p<* .01. ****p<*.001. Results for covariates can be obtained from authors.
