# Supplementary material for: A count of coping strategies: A longitudinal study investigating an alternative method to understanding coping and adjustment
Source: PLoS One. 2017 Oct 5;12(10):e0186057. doi: 10.1371/journal.pone.0186057 (PMC5642021; doi:10.1371/journal.pone.0186057)
Supplement: S3 Table — (DOCX) [file pone.0186057.s003.docx]

**Autoregressive Cross-Lagged Results for the Mean-based Model.**

|  | B | β | | SE | 95% CI |
| --- | --- | --- | --- | --- | --- |
| Positive Coping Mean1→Positive Coping Mean2 | 0.421 | 0.487 | *** | 0.024 | [.440, .533] |
| Positive Coping Mean1→Negative Coping Mean2 | -0.049 | -0.041 |  | 0.025 | [-.090, .008] |
| Positive Coping Mean1 →Depressive Symptoms2 | -0.003 | -0.002 |  | 0.025 | [-.051, .046] |
| Positive Coping Mean1→Suicidal Ideation2 | -0.016 | -0.049 |  | 0.025 | [-.099, .000] |
| Positive Coping Mean1→Emotion regulation2 | 0.033 | 0.024 |  | 0.024 | [-.022, .070] |
| Positive Coping Mean1→Self-esteem2 | 0.047 | 0.035 |  | 0.021 | [-.007, .077] |
| Positive Coping Mean1→Academic Achievement2 | 0.700 | 0.029 |  | 0.023 | [-.015, .074] |
| Negative Coping Mean1→Positive Coping Mean2 | 0.030 | 0.046 |  | 0.032 | [-.017, .109] |
| Negative Coping Mean1→Negative Coping Mean2 | 0.407 | 0.445 | *** | 0.028 | [.390, .500] |
| Negative Coping Mean1→Depressive Symptoms2 | 0.103 | 0.109 | *** | 0.030 | [.050, .167] |
| Negative Coping Mean1→Suicidal Ideation2 | 0.001 | 0.005 |  | 0.030 | [-.054, .065] |
| Negative Coping Mean1→Emotion Regulation2 | -0.086 | -0.081 | ** | 0.028 | [-.136, -.025] |
| Negative Coping Mean1→Self-esteem2 | -0.051 | -0.050 |  | 0.021 | [-.100, .001] |
| Negative Coping Mean1→Academic Achievement2 | -0.024 | -0.001 |  | 0.027 | [-.055, .052] |
| Depressive Symptoms1 →Positive Coping Mean2 | 0.035 | 0.053 |  | 0.038 | [-.022, .128] |
| Depressive Symptoms1 →Negative Coping Mean2 | 0.082 | 0.089 | * | 0.036 | [.019, .159] |
| Depressive Symptoms1 →Depressive Symptoms2 | 0.372 | 0.388 | *** | 0.035 | [.320, .456] |
| Depressive Symptoms1 →Suicidal Ideation2 | 0.049 | 0.196 | *** | 0.036 | [.125, .267] |
| Depressive Symptoms1 →Emotion Regulation2 | -0.052 | -0.048 |  | 0.034 | [-.114, .018] |
| Depressive Symptoms1 →Self-esteem2 | -0.039 | -0.037 |  | 0.031 | [-.098, .023] |
| Depressive Symptoms1 →Academic Achievement2 | -0.031 | 0.002 |  | 0.033 | [-.066, .062] |
| Suicidal Ideation1 → Positive Coping Mean2 | 0.008 | 0.003 |  | 0.028 | [-.051, .057] |
| Suicidal Ideation1 → Negative Coping Mean2 | -0.120 | -0.037 |  | 0.026 | [-.087, .014] |
| Suicidal Ideation1 → Depressive Symptoms2 | 0.099 | 0.029 |  | 0.026 | [-.022, .080] |
| Suicidal Ideation1 → Suicidal Ideation2 | 0.420 | 0.468 | *** | 0.024 | [.421, .515] |
| Suicidal Ideation1 → Emotion Regulation2 | 0.176 | 0.046 |  | 0.024 | [-.002, .094] |
| Suicidal Ideation1 → Self-esteem2 | -0.014 | -0.004 |  | 0.022 | [-.048, .040] |
| Suicidal Ideation1 → Academic Achievement2 | -1.224 | -0.019 |  | 0.024 | [-.065, .028] |
| Emotion Regulation1 → Positive Coping Mean2 | 0.071 | 0.121 | *** | 0.033 | [.057, .185] |
| Emotion Regulation1 → Negative Coping Mean2 | -0.029 | -0.035 |  | 0.031 | [-.096, .025] |
| Emotion Regulation1 → Depressive Symptoms2 | -0.033 | -0.039 |  | 0.031 | [-.100, .021] |
| Emotion Regulation1 → Suicidal Ideation2 | 0.002 | 0.011 |  | 0.031 | [-.050, .072] |
| Emotion Regulation1 → Emotion Regulation2 | 0.500 | 0.528 | *** | 0.027 | [.475, .580] |
| Emotion Regulation1 → Self-esteem2 | 0.050 | 0.054 | * | 0.027 | [.002, .106] |
| Emotion Regulation1 → Academic Achievement2 | 0.338 | 0.021 |  | 0.028 | [-.034, .076] |
| Self-esteem1 → Positive Coping Mean2 | 0.033 | 0.053 |  | 0.036 | [-.019, .124] |
| Self-esteem1 → Negative Coping Mean2 | -0.136 | -0.157 | *** | 0.034 | [-.223, -.090] |
| Self-esteem1 → Depressive Symptoms2 | -0.149 | -0.165 | *** | 0.034 | [-.232, -.099] |
| Self-esteem1 → Suicidal Ideation2 | -0.006 | -0.026 |  | 0.035 | [-.094, .042] |
| Self-esteem1 → Emotion Regulation2 | 0.113 | 0.112 | ** | 0.032 | [.049, .175] |
| Self-esteem1 → Self-esteem2 | 0.632 | 0.643 | *** | 0.027 | [.591, .695] |
| Self-esteem1 → Academic Achievement2 | 0.286 | 0.016 |  | 0.031 | [-.045, .077] |
| Academic Achievement1→ Positive Coping Mean2 | 0.002 | 0.039 |  | 0.026 | [-.011, .090] |
| Academic Achievement1 → Negative Coping Mean2 | -0.003 | -0.054 | * | 0.024 | [-.101, -.007] |
| Academic Achievement1 → Depressive Symptoms2 | -0.001 | -0.021 |  | 0.024 | [-.068, .027] |
| Academic Achievement1 → Suicidal Ideation2 | 0.000 | -0.024 |  | 0.025 | [.072, .024] |
| Academic Achievement1 → Emotion Regulation2 | 0.001 | 0.020 |  | 0.023 | [-.025, .065] |
| Academic Achievement1 → Self-esteem2 | 0.002 | 0.033 |  | 0.021 | [-.007, .074] |
| Academic Achievement1 → Academic Achievement2 | 0.778 | 0.719 | *** | 0.016 | [.687, .751] |

*Note.* β *=* standardized beta weights; B = unstandardized beta weights; *SE =* standard error, CI = standardized confidence intervals. Numbers 1 and 2 indicate Time 1 and Time 2,

respectively. **p* < .05. ***p<* .01. ****p<*.001. Results for covariates can be obtained from authors.
